# Addressing a Special Case of Zero-Crossing Range Adjustment Detection in a Passive Autoranging Circuit for the FBG/PZT Photonic Current Transducer

**DOI:** 10.3390/s25206311

**Published:** 2025-10-12

**Authors:** Burhan Mir, Grzegorz Fusiek, Pawel Niewczas

**Affiliations:** Department of Electronic and Electrical Engineering, University of Strathclyde, Glasgow G1 1XQ, UK; g.fusiek@strath.ac.uk (G.F.); p.niewczas@strath.ac.uk (P.N.)

**Keywords:** autoranging, zero crossing, fiber Bragg grating, piezoelectric transducer, photonic current transducer

## Abstract

This paper analyses a special case in evaluating the passive autoranging (AR) technique that dynamically extends the measurement range of a fiber Bragg grating/piezoelectric transducer (FBG/PZT) operating with a current transformer (CT) to realize a dual-purpose metering and protection photonic current transducer (PCT). The technique relies on shorting serially connected burden resistors operating with the CT, using MOSFET switches that react to a changing input current to extend measurement range. The rapid changes in the voltage at the FBG/PZT transducer that are associated with the MOSFET switching are then used on the FBG interrogator side to select the correct measurement range. However, when the MOSFET switching in the AR circuit occurs near the zero-crossing of the input current, the rapid changes in the voltage presented to the FBG/PZT no longer occur, rendering the correct range setting at the interrogator side problematic. The basic switching detection algorithm based on voltage derivative (dV/dt) thresholds proposed in the previous research is not sufficiently sensitive in these conditions, leading to incorrect range selection. To address this, a new detection algorithm based on temporal slope differencing around the zero-crossing is proposed as an additional detection mechanism for these special cases. Thus, the improved hybrid algorithm additionally computes the derivative dV/dt at the FBG/PZT voltage signal within a focused 6 ms temporal window centered around the zero-crossing point, a 3 ms window before and after each zero-crossing instance. It then compares the difference between these two values to a predefined threshold. If the difference exceeds the threshold, a switching event is identified. This method reliably detects even subtle switching events near zero crossings, enabling the accurate reconstruction of the burden current. The performance of the improved algorithm is validated through simulations and experimental results involving zero-crossing switching scenarios. Results indicate that the proposed algorithm improves MOSFET switching detection and facilitates reliable waveform reconstruction without requiring additional hardware.

## 1. Introduction

Precise voltage and current measurements are essential for effective metering and protection in electrical power systems, as mandated by IEEE and IEC standards [1]. Traditional iron-core current transformers (CTs) and voltage transformers (VTs) are commonly used for their proven reliability. However, they come with notable drawbacks, such as large size, heavy weight, high installation costs, and safety concerns related to galvanic coupling and oil-filled insulation systems [2,3].

In response to these limitations, fiber-optic current sensors (FOCS), a subset of non-conventional instrument transformers (NCITs), have emerged as practical alternatives. NCITs offer considerable advantages, including reduced size, enhanced safety, decreased environmental impact, increased operational bandwidth, and expanded dynamic range [4,5,6].

Optical sensing technologies, notably fiber-optic current sensors leveraging the Faraday effect, continue to advance due to their compact, lightweight design, broad bandwidth, high accuracy, electromagnetic immunity, and built-in galvanic isolation. While they can reach high precision (±0.1%) for both AC and DC measurements, their wider commercial use is hindered by relatively high costs, sensitivity to temperature changes, and vulnerability to mechanical vibrations. Furthermore, these sensors are devoid of essential functionalities such as passive multiplexing and remote interrogation, which are indispensable for wide-area monitoring, protection, and control (WAMPAC) applications.

To address these issues, the authors developed a range of optical current sensors combining the fiber Bragg gratings and piezoelectric transducers (FBG/PZT) to measure voltage across traditional CT burdens or Rogowski coils to form an FBG/PZT-based photonic current transducer (PCT). These sensors have the potential to meet strict IEC 5P protection class standards.

However, the FBG/PZT-based PCT has inherent limitations in the dynamic range and measurement accuracy compared to fiber-optic current sensors used as non-conventional instrument transformers [6,7,8]. Meeting the standards for 0,2 S metering, ideally combined with the 5P20 protection class, each with strict demands for measurement accuracy and dynamic range, continues to be a significant challenge for the proposed FBG/PZT-based PCT technology [9].

To address this issue, the authors developed passive autoranging (AR) circuits utilizing serially connected 1 Ω and 16 Ω burden resistors, along with MOSFET switches, allowing to significantly expand the PCT measurement range to ensure compliance with both metering (0,2 S) and protection (5P20) standards through a single device. Initial laboratory results confirmed that the passive autoranging circuit reliably responded to thresholds set at 130% of the rated current for metering and at 22 times the nominal current for protection. The implemented switching algorithm accurately reconstructed burden currents from optical signals recorded by the FBG interrogation system, confirming the possibility of achieving dual-class (metering and protection) functionality within a single photonic current transducer [10,11]. Furthermore, the circuit showed a fast response time of under 4 ms, meeting standard power system protection requirements [10]. Additionally, durability tests have demonstrated that the AR circuit can withstand secondary CT currents of up to 100 A for over 1 s, meeting key secondary circuit validation criteria required by certain grid operators [12].

However, a notable challenge of reliably detecting MOSFET switching events that occur precisely at or very close to zero-crossing remains unresolved [11]. Although these events are infrequent, they are critical because the existing detection algorithm may fail due to minimal voltage variations at zero-crossing points, resulting in incorrect reconstructed current waveforms. This paper directly addresses this limitation by proposing and validating an enhanced zero-crossing switching detection algorithm. Using advanced signal processing techniques such as precise waveform differentiation, dynamic thresholding, and localized data averaging, the algorithm successfully identifies the minimum slope variations in the signal at or very close to zero-crossings. The performance of the new hybrid algorithm is validated on the experimental data that was captured during the MOSFET switch deactivation and on the simulated data emulating the zero-crossing event during the switch activation. The results confirm improved and reliable waveform reconstruction, highlighting the algorithm’s effectiveness and its potential to enhance the robustness and applicability of FBG/PZT-based PCT in modern smart grid infrastructures. The method represents a novel approach and, to our knowledge, has not been employed in any sensor technology before. The method may have a broader significance in similar photonic or electronic sensor systems that are designed to rely on passive autoranging at remote sensor locations.

## 2. Materials and Methods

In previous research [10,11], the authors presented a passive auto-ranging method that uses multiple series-connected burden resistors controlled by static MOSFET switches. These switches quickly short or reconnect resistors based on preset current thresholds, expanding the dynamic measurement range. The MOSFET switches operate passively through a comparator circuit, which is powered directly from the CT within a fraction of the 50 Hz cycle. This passive configuration enables immediate range adjustments, which are detected in real-time via optical signal analysis by an FBG interrogator. As a result, the system can accurately apply scaling factors, allowing one CT to fulfill both metering and protection class requirements simultaneously.

The photonic current transducer configuration includes a dual-class CT with two burden resistors, an autoranging circuit, an FBG/PZT low-voltage transducer (LVT), and an integrated protection circuit (PC). The LVT operates between −30 V and 120. It reaches maximum displacement about 4.8 μs after a voltage step. To prevent depolarization or damage to the piezoelectric stack, its operational voltage is limited to ±30 V. Voltage signals applied to the stack induce mechanical strain, which causes the FBG sensor’s wavelength to shift proportionally. This shift directly reflects the measured voltage.

## 3. Experiment

### 3.1. Experimental Setup, Results and Zero-Crossing Switching Limitations

**Figure 1:**
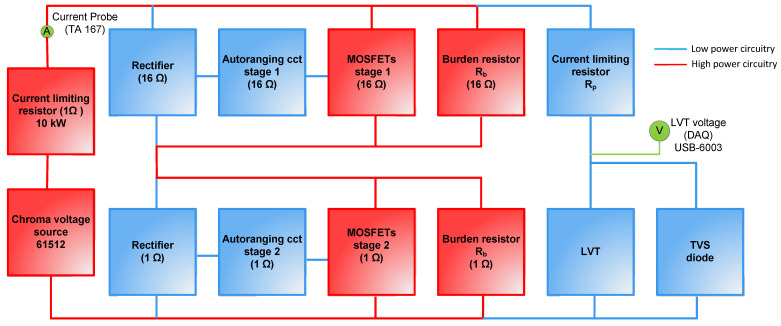
Block diagram of the experimental setup. Note the signal flow direction is from left to right.

The block diagram shown in Figure 1 depicts a PCT experimental evaluation setup for use with current transformers (CTs) with a rated secondary current of 1 A. The PCT under test aims to achieve both the IEC 0,2 S metering and 5P20 protection class accuracy within a single device. The configuration increases the PCT’s dynamic range and enables thermal protection against high currents. The AR circuit employs two burden resistors (16 Ω and 1 Ω) with the associated MOSFET switches, ensuring a quick response (~4 ms) to fault currents. At nominal current, the burden voltage is about 17 V, rising to 20.4 V at 1.2 A (IEC 0,2 S metering accuracy limit). When the 16 Ω resistor switches above 1.3 A, the LVT voltage drops to approximately 1.3 V, enabling accurate measurement up to 20 A (IEC 5P20 protection accuracy limit). An additional switch at 22 A shorts the 1 Ω resistor to prevent thermal overload. In summary, this setup delivers three modes: metering, protection, and thermal testing.

The circuit was tested experimentally with separate burden resistors equipped with dedicated switches, an LVT protected by a resistor–TVS assembly, and current supplied from a Chroma 61,512 source. Measurements were performed using a clamp-on current probe and an NI USB-6003 DAQ at a rate of 4 kHz/s.

The AR circuit shown in Figure 1 is integrated with an LVT, achieving the targeted response time of 4 ms and maintaining an ON state for 3–4 s to ensure proper fault clearance. It also performed reliably under thermal current conditions up to 100 A for one second. The switching logic functioned as intended, with the 16 Ω switching path activating at 1.3 A and the 1 Ω switching path at 22 A, preventing overheating and keeping the LVT voltage within ±30 V. Experiments confirmed the correct thresholds, timing, and safe operation across metering, protection, and thermal overload ranges. Switching events were identified by spikes in the derivative of the LVT output and processed through flip-flop logic to ensure proper signal scaling. The system performed as expected up to 100 A, as reported in detail in [11].

However, a special case was identified where the algorithm might struggle to detect AR circuit switching based solely on the rate of change in the optical signal. These events are improbable in real applications, but a theoretical possibility remains. One such scenario occurs when the switching, especially of the first switch (stage 1), happens exactly or very close to the zero-crossing of the input current signal (represented as a voltage at the LVT’s terminals). Although the switch may operate correctly and the signals can be scaled at the sensor as expected, the algorithm in its simplest form cannot recognize the switching moment based solely on the signal derivative. This is because such changes do not produce sufficient signal variation to be detected effectively by the derivative.

**Figure 2:**
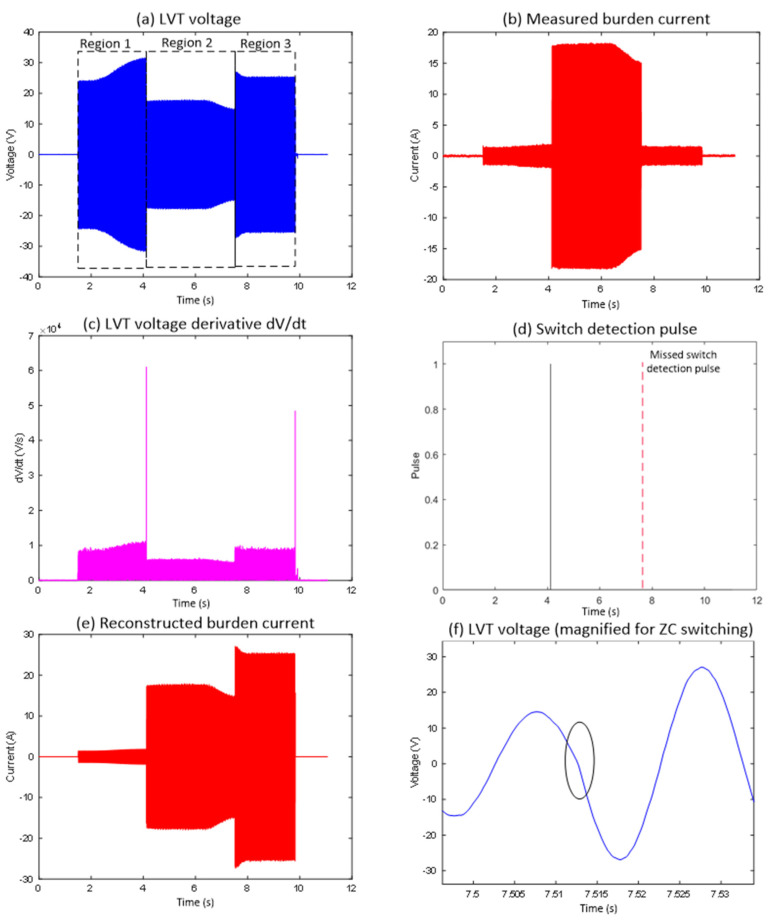
Experimental waveforms obtained for the case where MOSFET switching occurred near the input current zero crossing (Event 1).

**Figure 3:**
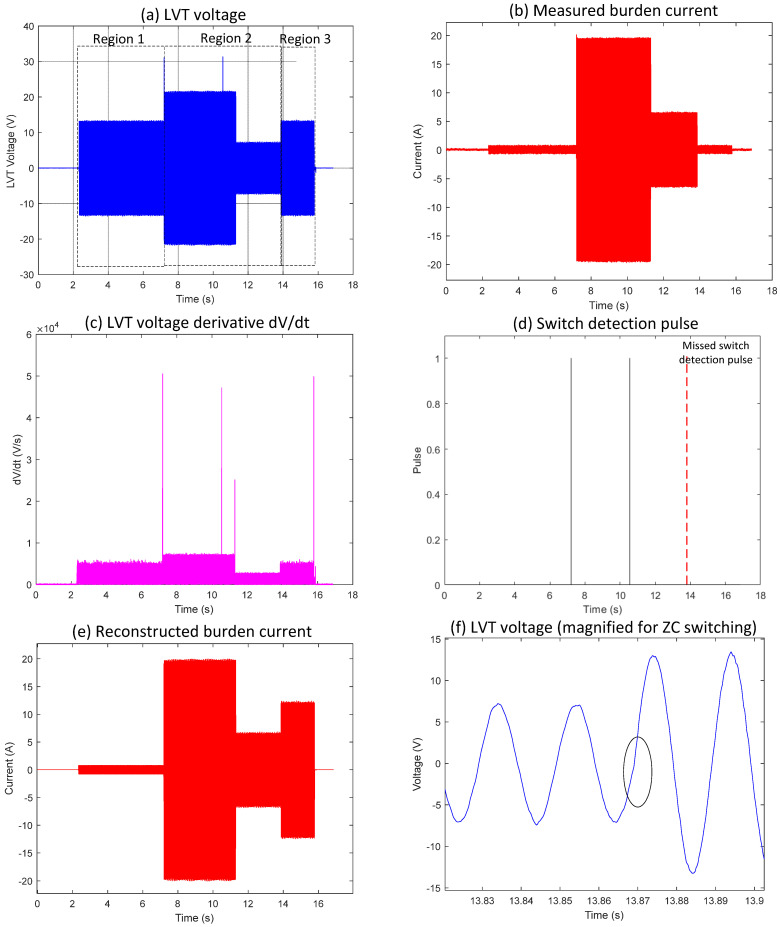
Waveform results obtained from the application of conventional algorithm to zero-crossing switching Event 2 (experiment).

**Figure 4:**
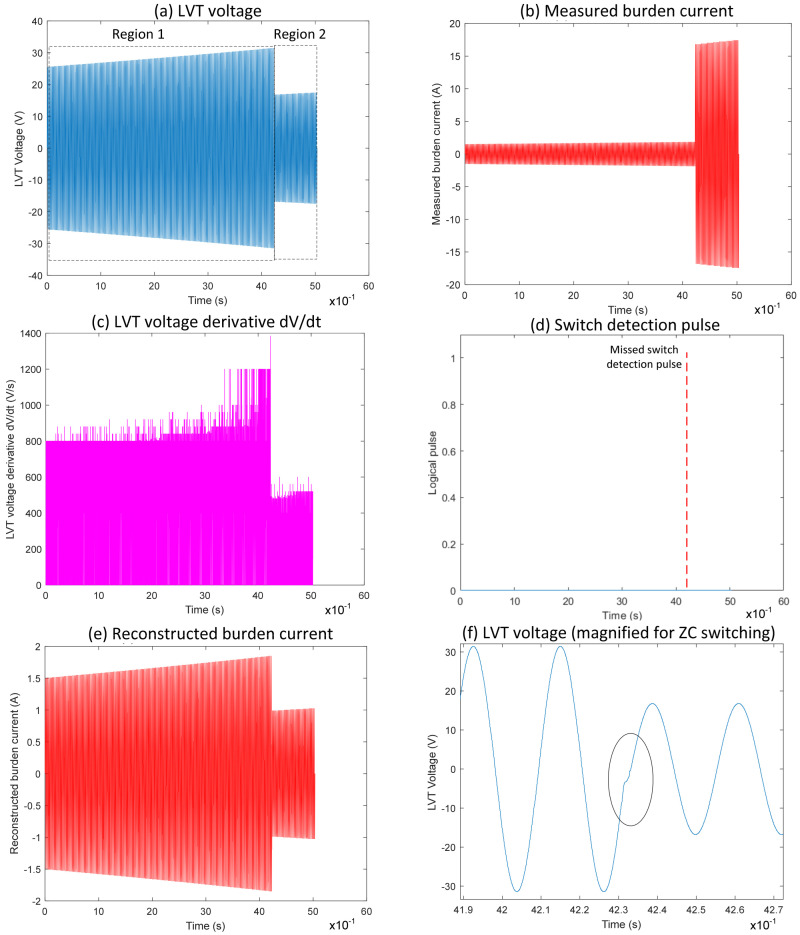
Waveform results obtained from the application of conventional algorithm to zero-crossing switching Event 3 (simulation).

Zoomed waveforms of the LVT voltage and the logic pulse derived from the LVT voltage when the first switch was deactivated are shown in Figure 2, Figure 3 and Figure 4. The switching occurred exactly or very close to the zero-crossing and was not detected by the derivative due to the absence of a rapid change in the LVT signal. As a result, the pulse indicating the change was not generated. In this case, the algorithm will continue to use incorrect scaling factors when calculating the measured current, potentially leading to false fault identification.

### 3.2. Zero-Crossing Switching Events (Basic Algorithm)

The zero-crossing switching events were recorded on two different occasions in electrical signals (LVT voltage) and once in a PSpice simulation. This is despite multiple trials, in the region of a hundred, to identify such events. The following subsections show that the basic algorithm, when applied to these specific recorded cases, was unable to identify the switching events in all three instances. This highlights the need for an improved algorithm to address this limitation.

#### 3.2.1. Zero-Crossing Switching Event 1 from Experiments

Figure 2 shows the first case that has been identified (Event 1), highlighting a range switching instance at the zero-crossing point when using the basic switching detection algorithm. Although the current threshold detection and MOSFET switching at the sensor location occurred as required, the reconstructed burden current, as shown in Figure 2e, differs significantly from the actual measured current in Figure 2b. This mismatch occurs because the MOSFET switch deactivates (enters the OFF state) exactly at the zero-crossing, as indicated in the highlighted section of Figure 2f. This event does not produce a large |dV/dt| change, resulting in the algorithm missing this switching event altogether (see the missing switch detection pulse indicated in Figure 2d in red dotted line). This is to be noted that the algorithm calculates and plots absolute values of dV/dt as seen in Figure 2c.

Regarding the autoranging process in detail, in Region 1 (Figure 2a), the LVT voltage increases gradually but remains within the ±30 V operational limit. During this time, the measured current follows the expected scaling relation I = V/17, which keeps both MOSFET switches turned off. When the LVT voltage reaches the activation threshold, Region 2 begins. Here, the first switch turns on, changing the burden current scaling to I = V/1, as shown by the rise in Figure 2b at around the 4 s mark. The algorithm correctly identified this switching event, as indicated by the detection pulse in Figure 2d. In Region 3, starting at around the 7.5 s mark, the switch turns off, and the scaling returns to I = V/17, as depicted in Figure 2b. However, the algorithm did not detect this second transition, and no pulse appears in Figure 2d for this event. As a result, the reconstruction logic continues to use the wrong scaling factor during this period, causing a noticeable discrepancy between the reconstructed and measured burden currents when comparing Figure 2b and 2e. The circled area in Figure 2f shows that the switching event occurred precisely at the zero-crossing, which results in an insufficient gradient in the derivative signal to trigger a detection response. This highlights a limitation of the detection method when switching events occur at zero-crossing points.

#### 3.2.2. Zero-Crossing Switching Event 2 from Experiments

Event 2, shown in Figure 3, demonstrates a case where switching happens at a zero-crossing point within the typical switching algorithm framework. As seen in Figure 3e, the reconstructed burden current differs from the actual measured current in Figure 3b because the MOSFET switch-off occurs at the zero-crossing point.

In Region 1 of Figure 3a, the LVT voltage remains steady within the ±30 V limit. The measured burden current in Figure 3b follows the expected scaling factor of I = V/17, indicating that both MOSFET switches are off and the full burden resistance is active. When Region 2 begins, the first switch turns on, changing the current scaling relation to I = V/1, as shown by the step change in Figure 3b. This switching event was correctly identified by the algorithm, with a corresponding detection pulse visible in Figure 3d. The second pulse at approximately 10.5 s represents the intermittent switching, during which the autoranging circuit enters a recovery mode, allowing the main capacitor to recharge and provide the necessary charge for the control circuit to operate. Region 3 marks the point where the switch turns off again, returning the scaling factor to I = V/17, as seen in Figure 3b. However, unlike the earlier transition, the algorithm did not detect this second switching event, and no switch detection pulses appear in Figure 3d. As a result, the reconstructed burden current in Figure 3e for this interval is incorrect compared to the measured signal. The encircled area in Figure 3f highlights the part of the LVT voltage where the switch-off took place precisely at the zero-crossing. Because the voltage derivative at this point shows minimal change, the algorithm did not recognize a distinct transition and thus did not generate a detection pulse. This explains the discrepancy between the reconstructed and measured current signals in the affected region.

#### 3.2.3. Zero-Crossing Switching Event 3 Form PSpice Simulation

Figure 4 illustrates Event 3, a case from PSpice simulation, which highlights the limitations of the conventional switching algorithm when a switching-on event coincides with the zero-crossing of the voltage signal during the first switch activation. The difference between the measured and reconstructed burden currents is evident when comparing Figure 4b,e. In Region 1 (Figure 4a), the LVT voltage increases gradually, staying within the ±30 V range. At the same time, the measured current follows the scaling factor I = V/17, consistent with both MOSFET switches being inactive during this period.

As the LVT voltage crosses the specified threshold, Region 2 begins with the first MOSFET turning on, which affects the scaling to I = V/1. However, this transition is not detected by the switching algorithm, as shown by the absence of a detection pulse Figure 4d. Consequently, the system continues using the previous scaling, leading to an inaccurate reconstruction of the burden current. The area impacted is further shown in Figure 4f, where the switching occurs very close to zero-crossing. Due to the minimal slope in the voltage derivative at this point, the algorithm fails to detect the event. This demonstrates that switching events near zero-crossings are challenging to detect with derivative-based thresholding, ultimately leading to incorrect current reconstruction following these transitions.

## 4. Zero-Crossing Switch Detection Algorithm

Although the autoranging technique proved effective during high-current events in the great majority of recorded cases, switching at zero-crossings created challenges for the basic algorithm. The minimal derivate variations due to MOSFETs switching occurring at or very close to zero-crossings of the LVT voltage/burden current prevented the basic algorithm from accurately detecting these events, leading to incorrect gain adjustments and incorrect current waveform reconstructions. As mentioned earlier, switching at zero-crossings is least likely to occur. During the experiments, we were unable to capture zero-crossing switching in the optical signals despite hundreds of tests. However, two such events were recorded as the LVT voltage signals, largely by chance. Similarly, as described in Section 3, one such event was also identified through a simulation. As explained, the basic range detection algorithm failed to identify the zero-crossing switching, causing large errors in the reconstructed burden current. This motivated us to develop and design a new algorithm capable of detecting zero-crossing switching events.

### 4.1. Design of Improved Algorithm

The proposed algorithm is designed to reliably reconstruct the burden current from the LVT voltage signal, with special focus on detecting switching events near zero-crossings. Instead of relying on amplitude or RMS-based methods, which lack resolution within the 4 ms response time requirement for the autoranging system, this method emphasizes transient features in the LVT waveform.

**Figure 5:**
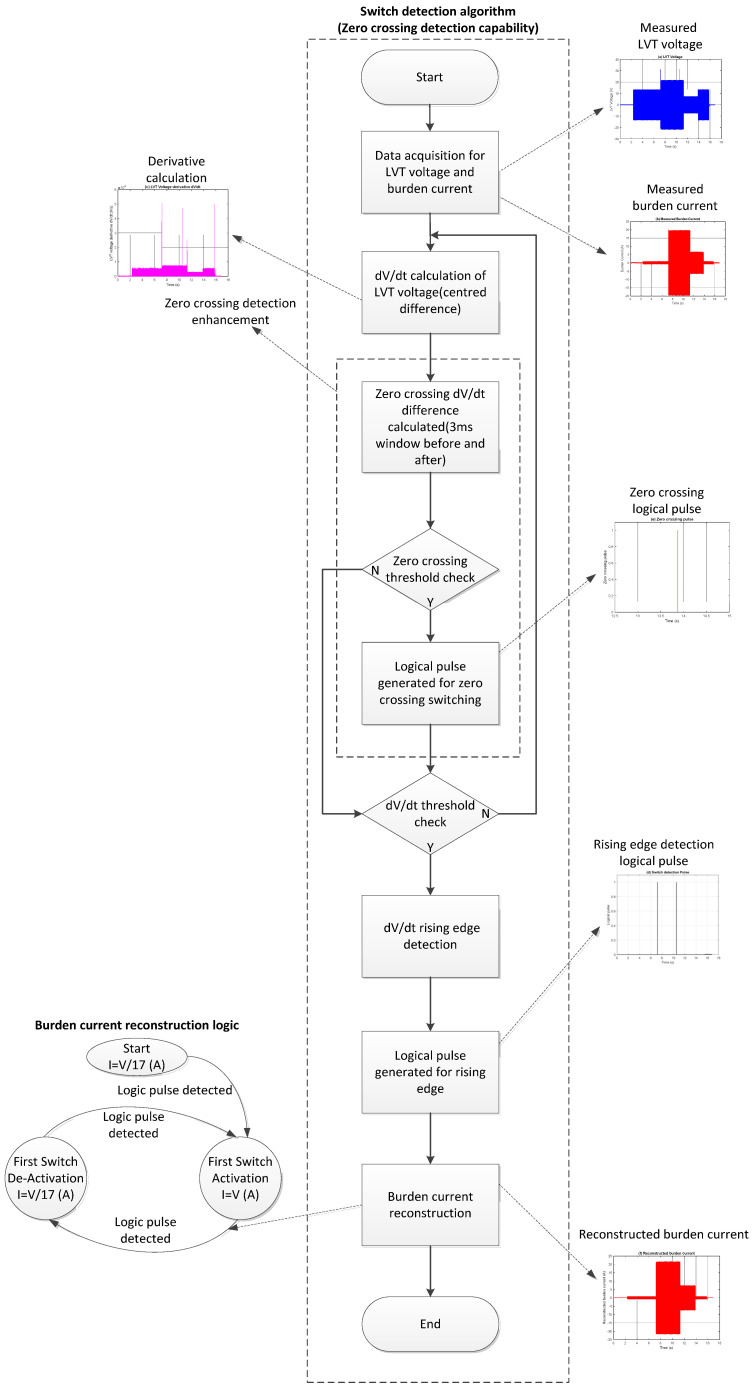
Functional diagram of zero-crossing switch detection algorithm.

The algorithm, as shown as a functional diagram in Figure 5, begins by calculating the derivative of the LVT voltage using a centered difference scheme. Under normal conditions, the derivative transitions or spikes remain below a certain level. MOSFET switching causes abrupt changes in the LVT voltage signal, which appear as significant spikes in the derivative profile. When these spikes exceed a specified threshold, they are used to generate logic pulses that indicate switching events.

To identify spikes that occur during zero-crossings where the derivative may be minimal, a focused 6 ms temporal window centered around the zero-crossing point is extracted. The average derivative is calculated separately for the pre- and post-zero-crossing intervals, and their absolute difference is compared to a reduced threshold window, optimized for low-slope detection. If this condition is met, the algorithm records a switching event and outputs a corresponding logic signal.

Outside zero-crossing regions, a higher threshold is used for detection because switching outside these regions causes significant spikes that are easily detectable. This threshold is chosen based on a previous inspection of the LVT derivative signal and is calibrated to identify more abrupt switching transitions reliably. Each time this threshold is crossed, a rising-edge-triggered logic pulse is generated, indicating either the activation or deactivation of a MOSFET. These logic signals serve as the timing markers for the sensor interrogation system that are needed to select the appropriate scaling factors during current reconstruction.

The reconstruction method uses the sequence of switching events to determine the proper scaling factor at each moment. When both switches are open, the burden current is scaled by a factor of 1/17. Once the first switch engages, the 16 Ω burden is bypassed, and the scaling factor is updated to 1. When the switch returns to its open state, the original scale is restored. This logic repeats dynamically based on real-time switching conditions. The implementation was carried out in MATLAB R2021b and tested using logged experimental data from the AR system, sampled at 4 kHz, as well as similar data from PSpice simulation. With known burden resistor values and precise switching times, the reconstructed current waveform closely matches the measured signal. This method provides increased robustness in detecting subtle signal transitions, particularly near zero-crossings, while minimizing false detections.

### 4.2. Zero-Crossing Switching Event 1 (Experiment)

**Figure 6:**
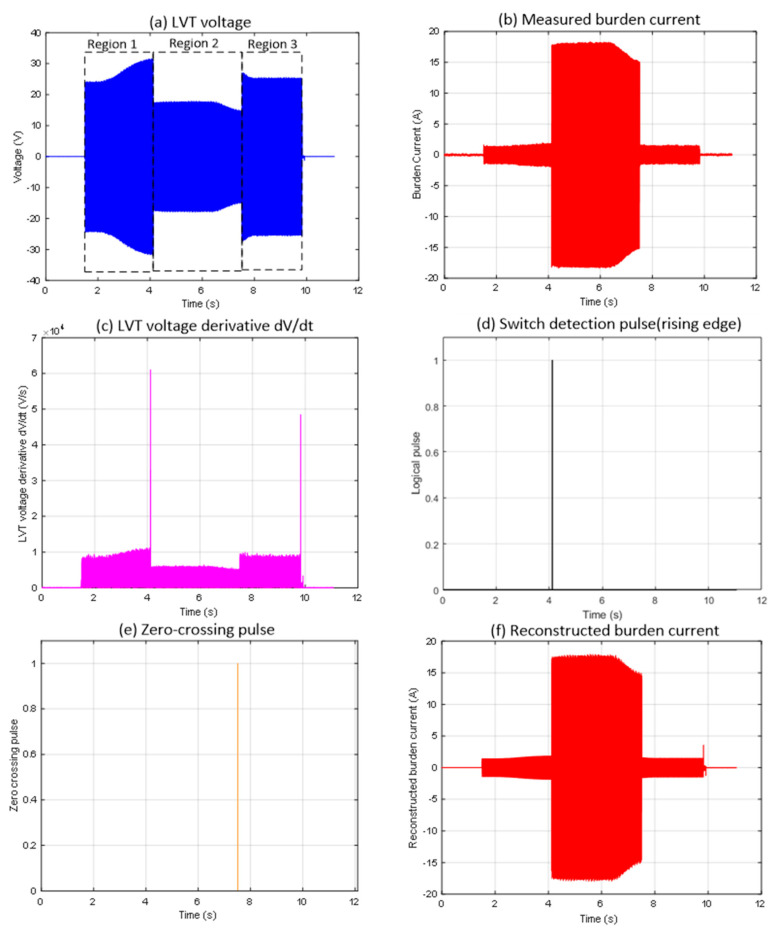
Waveform results obtained from the application of advanced zero-crossing switch detection algorithm to zero-crossing switching Event 1 (experiment).

Figure 6 illustrates Event 1, demonstrating the effective operation of the proposed detection algorithm in identifying a switching event that occurs precisely at the zero crossing. Unlike the conventional algorithm, which was unable to detect such events, the improved algorithm successfully captures the event, allowing for the accurate reconstruction of the burden current. As shown in Figure 6f, the reconstructed current waveform aligns closely with the measured current in Figure 6b, confirming the effectiveness of the algorithm.

In Region 1 (Figure 6a), the LVT voltage increases gradually, remaining within the ±30 V range, and the burden current follows the expected scaling of I = V/17, indicating the inactive state of both MOSFETs. Once the voltage reaches the switching threshold, Region 2 begins with the activation of the first MOSFET, which alters the effective burden and shifts the current scaling to I = V/1. This change is clearly visible in the measured and reconstructed current and was correctly identified by the algorithm, as shown by the detection pulse in Figure 6d. In Region 3, the switch turns off at the zero-crossing. Thanks to the algorithm’s targeted derivative analysis near zero-crossing points, this deactivation is also accurately detected. The corresponding logic pulse appears in Figure 6e. Consequently, the burden current reconstruction in Region 3 uses the correct scaling factor, resulting in a high level of consistency between the measured and reconstructed signals, as demonstrated in Figure 6b,f. This example illustrates the enhanced temporal resolution and autorange decision accuracy of the proposed method, particularly in scenarios where traditional approaches struggle to capture critical switching events.

### 4.3. Zero-Crossing Switching Event 2 (Experiment)

**Figure 7:**
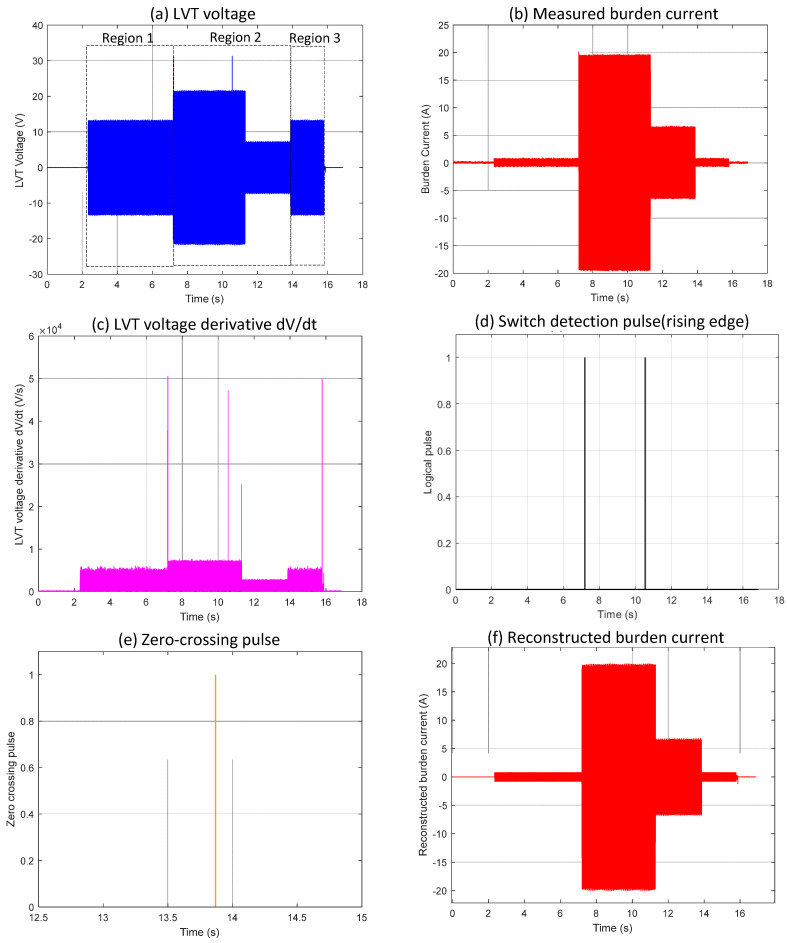
Waveform results obtained from the application of advanced zero-crossing switch detection algorithm to zero-crossing switching Event 2 (experiment).

Figure 7 illustrates Event 2, where a switching transition occurs at the voltage zero-crossing and is accurately detected by the improved detection algorithm.

The reconstructed burden current using this method closely matches the measured current waveform, as shown by the comparison between Figure 7b,f. In Region 1 (Figure 7a), the LVT voltage remains stable within the ±30 V safety limit. During this period, the current scales according to I = V/17, which is consistent with both MOSFETs being inactive and the full burden resistance of 17 Ω present in the circuit.

Region 2 starts when the first switch being activated, reducing the effective resistance and updating the scaling factor to I = V/1. This change is visible in the measured current and was correctly identified by the algorithm, as shown by the detection pulse in Figure 7d, where intermittent switching behavior is also noted. Region 3 marks the switch deactivation point, which occurs precisely at the zero-crossing, an event that, without targeted detection, would be difficult to spot due to the minimal signal slope. However, the new algorithm’s specialized hybrid detection, involving the zero-crossing switching case, successfully captures this event, as indicated by the corresponding pulse in Figure 7e. As a result, the correct scaling factor is used and the reconstructed burden current shown in Figure 7f closely matches the measured current in Figure 7b, confirming the effectiveness of the new detection method in resolving switching events at zero-crossings.

### 4.4. Zero-Crossing Event 3 (Simulation)

**Figure 8:**
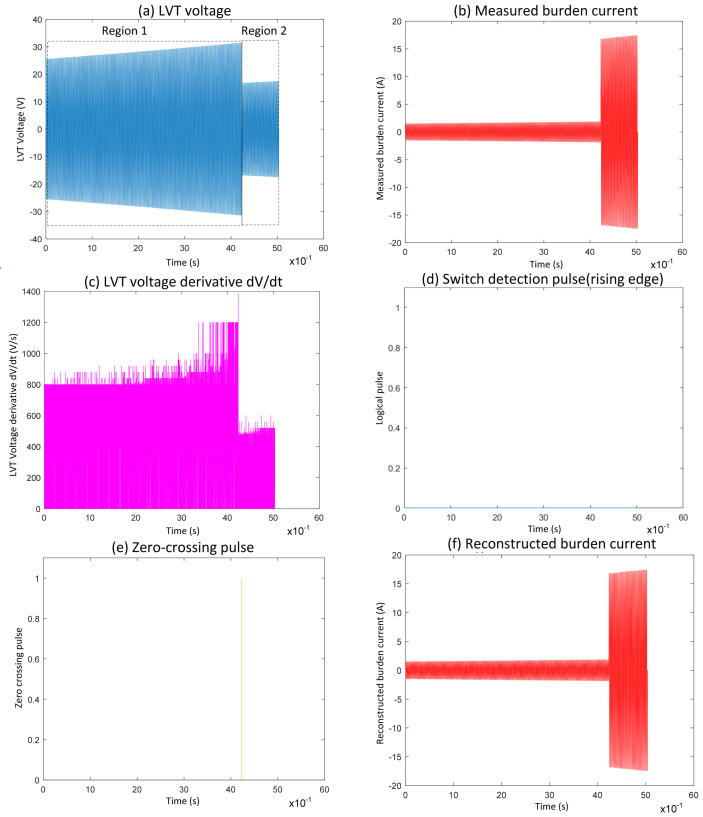
Waveform results obtained from the application of advanced zero-crossing switch detection algorithm to zero-crossing switching Event 3 (simulation).

Figure 8 shows Event 3, based on PSpice simulation results, highlighting the limitations of traditional switching algorithms when the switching-on event aligns with the zero-crossing of the LVT voltage signal during the first switch activation. In this case, the proposed algorithm shows its improved detection ability. A comparison between Figure 8b,f reveals that the reconstructed burden current accurately matches the measured waveform, confirming that the switching event at zero-crossing was correctly detected and processed.

During Region 1 (Figure 8a), the LVT voltage increases steadily while remaining within the ±30 V range. The measured current shows a scaling relationship of I = V/17, consistent with the inactive state of both MOSFET switches. When the LVT voltage reaches the switching threshold, Region 2 begins, and the first MOSFET turns on. This event, occurring at the zero-crossing, changes the current scaling to I = V/1. Despite the flatter slope of the voltage derivative at this point, the algorithm successfully detects the transition, as evidenced by the switching pulse shown in Figure 8e. This detection enables the reconstruction logic to apply the correct scaling factor in real-time, resulting in accurate burden current estimation throughout the interval.

This case further confirms the algorithm’s ability to identify switching activity in low-gradient conditions near the zero-crossing point, a scenario that the previously used basic method may struggle to resolve. The comparison of the reconstructed current in Figure 8f and the measured current in Figure 8b demonstrates both the accuracy and dependability of the algorithm in situations where signal features are least apparent.

## 5. Discussion

The improved zero-crossing switch detection algorithm effectively addresses a key limitation in the existing method used in passive autoranging circuits for FBG/PZT photonic current transducers. This enhancement ensures correct measurement scaling, which is vital for meeting dual-class requirements in metering and protection applications. The earlier technique which primarily relied on identifying voltage discontinuities or abrupt changes in voltage slopes, often fail to detect switching events that occur precisely at or near zero crossings due to minimal voltage variations. The new algorithm addresses this problem by carefully comparing voltage slope differences immediately before and after the zero-crossing point, thereby significantly enhancing detection sensitivity. As previously discussed, switching events that coincide with zero-crossings are very rare and were not observed in optical signal measurements despite extensive testing. However, on two separate occasions, such events were detected in the electrical signals, specifically across the LVT voltage terminals and burden current signals. Through two experimental setups and one simulation scenario, the algorithm consistently identified switching events that the basic method missed. This improved hybrid detection ability results from calculating and analyzing voltage slope derivatives within narrow time frames around each zero-crossing. Results demonstrate that even minor signal variations are successfully detected, ensuring accurate application of scaling factors and reliable reconstruction of burden currents. Importantly, this algorithm requires no additional hardware beyond what is already available, highlighting its practicality and cost-effectiveness. Its capacity to detect rare but significant zero-crossing events enhances the reliability and robustness of photonic current transducers, promoting broader adoption in modern electrical grid monitoring and protection systems.

## 6. Conclusions and Future Work

This study introduces a novel algorithm specifically designed to overcome the limitations of the existing method in detecting zero-crossing events in passive autoranging circuits for FBG/PZT photonic current transducers. By using precise temporal slope comparisons, the algorithm reliably detects zero-crossing switching events, significantly improving the accuracy of burden current reconstruction. In all tested cases, the algorithm accurately detected both switching and non-switching events, including those at or near voltage zero-crossings. This precise event detection enabled the correct application of scaling factors, resulting in the accurate reconstruction of the burden current in all scenarios. The algorithm was developed and implemented in MATLAB and applied to two experimentally recorded events as well as one additional event generated through PSpice simulation. Experimental and simulation tests verified the algorithm’s effectiveness, reliably identifying and reconstructing the burden current, which was previously inaccurately determined, through the conventional technique. Overall, the improved detection ability greatly increases measurement accuracy, reliability, and the practical usability of photonic current transducers. The results of this study demonstrate a strong potential for integrating this detection algorithm into existing photonic sensing systems, thereby meeting stringent industry standards for accuracy, responsiveness, and reliability. This study focuses on analyzing recorded data to validate the algorithm’s detection and reconstruction capabilities after data collection process. Future research will focus on implementing this detection algorithm in real-time systems, allowing continuous monitoring and immediate adjustment of scaling factors. Testing the algorithm in live conditions will offer further validation across different operating environments. Detailed assessments of detection accuracy, noise resilience, and operational efficiency will provide valuable insights for future improvements. Additional efforts will also focus on capturing zero-crossing switching events in optical measurements, thereby broadening the algorithm’s validation to encompass both electrical and optical signals. This comprehensive approach will significantly improve the practical deployment of FBG/PZT photonic current transducers in advanced smart grid systems. Although the algorithm has only been applied to electrical data so far, it can be fully adapted for use with optical measurements if a zero-crossing switching event is captured in the optical signal. Ongoing experimental efforts are focused on recording such events in optical signals. Future efforts will involve embedding the algorithm into a real-time measurement system to evaluate its performance under live conditions. Further development will also include quantitatively evaluating sensor accuracy across various current ranges and fault scenarios.

## Data Availability

All data underpinning this publication are openly available from the University of Strathclyde KnowledgeBase at https://doi.org/10.15129/e9a4ac0e-8e25-4ab6-8b0a-5de63e5e0bdc (accessed on 10 October 2025).
